# Comprehensive Assessment of Degradation Behavior of Aspirin and Atorvastatin Singly and in Combination by Using a Validated RP-HPLC Method

**DOI:** 10.3797/scipharm.1210-19

**Published:** 2012-12-11

**Authors:** Omkar Sherikar, Priti Mehta

**Affiliations:** Department of Pharmaceutical Analysis, Institute of Pharmacy, Nirma University, Sarkhej-Gandhinagar Highway, Ahmedabad, Gujarat, 382481, India.

**Keywords:** Aspirin, Atorvastatin, Chemical incompatibility, Degradation profile, Drug combination, HPLC, Acetylsalicylic acid

## Abstract

A fixed-dose combination of atorvastatin and aspirin is widely used for the treatment of myocardial infarction. The present work describes a comprehensive study of the stress degradation behavior of atorvastatin and aspirin alone as well as in combination of 1:1 and 1:7.5 ratios, respectively, as per ICH guidelines. The degradation products of aspirin as well as atorvastatin were successfully separated by a developed simple, selective, and precise stability-indicating reversed-phase HPLC method. Chromatographic separation was achieved on the Phenomenex Luna analytical column, 150 mm × 4.6 mm, 5μm. The mobile phase consisted of 0.1% glacial acetic acid in water and acetonitrile in the ratio of 50:50 v/v at a flow rate of 1.0 ml/min. UV detection was performed at 246 nm. The extent of degradation was significantly influenced when both of the drugs were present in combination. Stress degradation behavior of atorvastatin was highly influenced by aspirin under acid hydrolysis, thermal degradation, and oxidative stress conditions. Similarly, the stress degradation behavior of aspirin was affected by atorvastatin especially under neutral hydrolysis, thermal degradation, and oxidative stress conditions. Additionally, the combination ratio of aspirin and atorvastatin also influenced the percentage degradation of each other. A mixture of aspirin and atorvastatin was also analyzed after a one-month stability study at 40 °C and 75% RH. All the results indicate chemical incompatibility of both aspirin and atorvastatin if present in combination.

## Introduction

The main cause of myocardial infarction (MI) is the interruption of the blood supply to the heart, which is most commonly due to the blockage of a coronary artery due to a hyperlipedemic condition. For the mitigation of such a condition, lipid lowering agents are widely used alone or in combination with other antihypertensive agents or with aspirin. Several fixed-dose combinations (FDCs) have been approved by the Food and Drug Administration (FDA) along with a combination of ATR and ASP due to their effectiveness against this condition [1, 2].

**Fig. 1:**
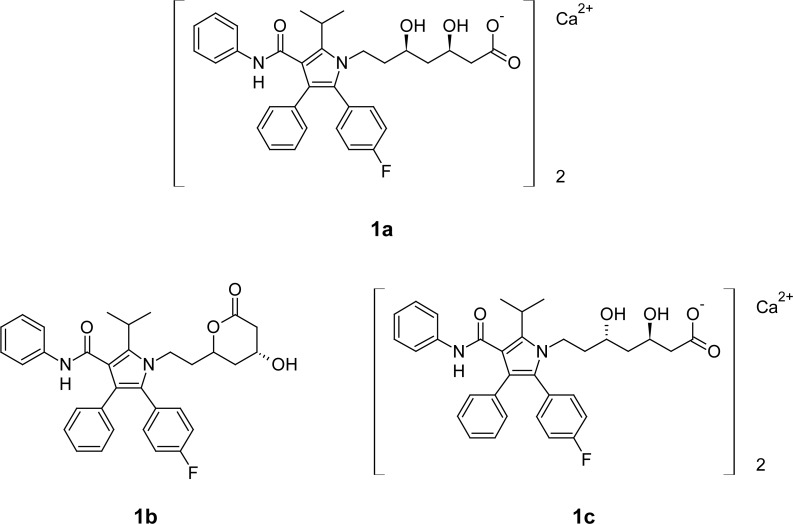
Chemical structure of atorvastatin calcium (**1a**) and its major degradation products atorvastatin lactone (**1b**) and atorvastatin diastereomer (**1c**).

Atorvastatin calcium (ATR) is an antihyperlipidemic agent. It lowers lipid levels by inhibiting HMG-CoA reductase, a rate-limiting enzyme in cholesterol biosynthesis in the liver, thus reducing the cholesterol content in hepatocytes. ATR is chemically (3*R*,5*R*)-7-[2-(4-fluorophenyl)-3-phenyl-4-(phenylcarbamoyl)-5-(propan-2-yl)-1*H*-pyrrol-1-yl]-3,5-dihydroxy-heptanoic acid (Fig. 1a) [3, 4].

**Fig. 2:**
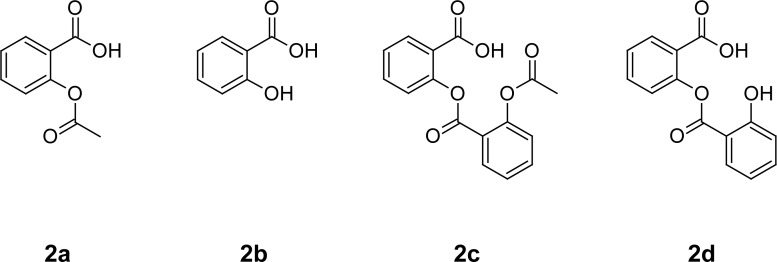
Chemical structure of aspirin (**2a**) and its degradation products salicylic acid (**2b**), acetylsalicylsalicylic acid (**2c**), and salicylsalicylic acid (**2d**).

Aspirin (ASP) is a cyclooxygenase inhibitor and is commonly used as an analgesic, anti-inflammatory, and antipyretic. Additionally, it also has an antiplatelet effect, hence, it is also used for the prevention of heart attacks. ASP is chemically 2-(acetyloxy)benzoic acid (Fig. 2) [3, 4]. The physicochemical, pharmacological, and pharmacokinetic characteristics of both ASP and ATR are well investigated [5–8].

When ASP and ATR are used in combination, thromboxin-A_2_ (TXA_2_)-dependant aspirin resistance is reduced. This mechanism is especially helpful to patients with acute myocardial infarction and persistent platelet TXA_2_ production. In addition, ATR also reduces lipid levels [9].

In the Indian market, FDC capsule formulations are available from various manufacturers like Sun Pharmaceuticals Ltd. (AZTOR ASP 75), Zydus Healthcare Ltd. (ASP ATORVA 75), and Piramal Healthcare Ltd. (STATOR ASP 75) having strengths of 10 mg and 75 mg of ATR and ASP respectively.

However, when FDCs are formulated, chemical incompatibility is an important factor influencing drug stability. Interactions may take place either among different active ingredients or active ingredients and excipients within the formulation. Chemical instability always leads to the decomposition of actives present in the formulation [10, 11].

The chemical structure reveals that ASP is an ester moiety, and very susceptible to hydrolysis under different hydrolytic conditions. Decomposition of ASP into salicylic acid (SA) is well-known [5, 12, 13]. On the other hand, ATR is an organic acid with a pK_a_ of 4.46 and it is an acid-labile moiety which gets converted into lactone under acidic conditions [14–16].

Several analytical methods have been reported for the estimation of ASP and ATR in the presence of each other as well as along with other drugs [17–23]. A literature survey revealed that only one UPLC stability-indicating assay method is reported for the combination of ASP and ATR in capsule dosage form [24].

So far, no reports are available regarding the comprehensive study on degradation behavior of ATR and ASP in the presence of each other. From the reported data of the degradation profile of both drugs, it was essential to evaluate the chemical stability of ASP and ATR in combination. It was hypothesized that ATR may get degraded in the presence of ASP, as ATR is an acid-labile moiety and ASP is acidic in nature.

Stress testing is an important tool for the prediction of stability-related problems in pharmaceutical products. Therefore, in the present paper, a stress degradation study of ATR and ASP was performed in combination of each other as per ICH guidelines and degradation behavior of both drugs was monitored precisely. Additionally, a one month stability test of a mixture of ATR and ASP was also evaluated to further confirm the chemical incompatibility of these two drugs [25].

## Materials and Methods

### Drug and Chemicals

Pure ATR was kindly gifted by Dr. Reddys Ltd, (Hyderabad, India), certified to contain 99.1%, and used without further purification. Standard ASP was purchased from Sigma Aldrich (India) with the stated purity of 99.8%. Salicylic acid was purchased from SD Fine Chemicals, Mumbai, with the stated purity of 99.0%. Atorvastatin lactone was procured from Varda Biotech Pvt. Ltd, Mumbai, India. FDC of ATR and ASP was purchased from the local market with each capsule containing 10mg of ATR and 75mg of ASP respectively.

HPLC grade acetonitrile was purchased from Merck (Mumbai, India). HPLC grade glacial acetic acid and dimethyl sulfoxide (DMSO) were procured from CDH, New Delhi. 0.45 μm nylon filters were used for sample filtration. Mili-Q water was used throughout the study.

### Instrumentation and Chromatographic Condition

Analysis was carried out on the HPLC system (Jasco, Japan) consisting of the binary pumps-Jasco PU-2080 and solvent mixing module-Jasco MX-2080, equipped with the photodiode array (PDA) detector MD-2015 Plus (Jasco Japan). It also consisted of the Rheodyne manual injector having a 20μL capacity. Data collection and data processing were accomplished by using BORWIN-PDA software.

Separation was achieved on the Phenomenex Luna analytical column, 150 mm × 4.6 mm, 5μm. The mobile phase consisted of 0.1% glacial acetic acid in water and acetonitrile in the ratio of 50:50 v/v. The mobile phase was filtered through a 0.45μm nylon filter and degassed prior to use. An equal amount of acetonitrile and water was mixed and used as a diluent throughout the study.

## Experimental

### Preparation of Standard Stock Solution

The standard stock solutions, 1000 μg/mL each of ATR and ASP, were prepared individually in methanol.

### Preparation of samples for the Forced Degradation Study

Forced degradation of ASP and ATR, individually as well as in combination, was carried out as per ICH guidelines. Both ASP and ATR were forcibly degraded under different stress conditions such as hydrolytic, oxidative, thermal, and photolytic degradation.

To perform the degradation study in combination, two ratios 1:1 and 1:7.5 were selected for ATR and ASP, respectively. All degradation samples were prepared in DMSO and the respective stressor in the ratio of 50:50 v/v. Stress samples were prepared by taking a concentration of 2.5 mg/mL of each drug. Hydrolytic degradation was carried out in 0.1N HCl, 0.1N NaOH, and distilled water by dissolving ASP and ATR in 25 mL of DMSO and then the volume of this solution was made up to 50 mL with 0.1N HCl, 0.1N NaOH, and distilled water respectively. All prepared samples were refluxed at 80 °C for 3 hours.

For oxidative stress, 3%v/v hydrogen peroxide was used. For both ASP and ATR, 2.5 mg/mL samples were prepared by dissolving the drug in 25 ml of DMSO and then the volume was made with 3%v/v hydrogen peroxide solution and samples were kept at room temperature for seven days. After completion of stress exposure, samples were neutralized and further diluted to get a concentration of 50 μg/mL each of ATR as well as ASP, respectively, for HPLC analysis. In the case of a 1:7.5 ratio, 50 μg/mL concentration of ATR was selected and this correspondingly gave 375 μg/mL of ASP. Thermal degradation was carried out in a solid state in a controlled temperature oven at 80 °C for 48 hours. For photodegradation, solid samples were spread uniformly in a petridish. Then samples were exposed to bright, sunny days from 11 am to 5 pm for two days. So the samples were exposed to sunlight for a total of 12hrs.

In addition, ASP and ATR, alone as well as in combination, were kept for solid-state stability studies for one month at 40°C and 75% RH in a stability chamber to extrapolate the probable chemical incompatibility between these two drugs. Stock solutions of all solid samples were prepared in methanol. Appropriate aliquots were further diluted to get 50 μg/mL each of ATR and ASP, respectively, for HPLC analysis.

### Method Validation

The developed stability-indicating assay method was validated by assessing various validation parameters such as linearity, precision, accuracy, specificity, limit of detection and quantification, and solution stability, as prescribed by ICH guidelines [26].

### Linearity

The calibration curves of ASP and ATR were constructed over a concentration range of 1–80 μg/mL and 1–60 μg/mL respectively. Different sets of six concentrations were prepared and 20 μL of each solution were injected in triplicate under the operating chromatographic conditions.

### Method Precision

Repeatability (method precision) was assessed by analyzing the capsule samples six times having a concentration of 60 μg/mL and 8 μg/mL for ASP and ATR respectively.

The intraday and interday precision of the proposed method was determined by estimating the corresponding responses three times on the same day as well as on three different days by taking three different concentrations of ASP and ATR (10, 20, and 40 μg/mL). The result of the precision study was evaluated in terms of % RSD.

### Specificity

The specificity of the method was assessed by checking the peak purity of both ASP as well as ATR. Interference from degradation peaks in the estimation of ASP as well as ATR was also checked.

### Accuracy

The accuracy of the method was determined by estimating recoveries of ASP and ATR by the standard addition method at three different levels (80,100, and 120 %) to the preanalyzed capsule formulation. A known amount of standard ASP (24, 30, and 36 μg) and ATR (3.2, 4, and 4.8 μg) were added to the preanalyzed sample solution containing 30 μg/mL of ASP and 4 μg/mL of ATR respectively. The accuracy of the method was determined by comparing the amount recovered and the actual amount spiked.

### Robustness

The robustness of the method was evaluated by analyzing the same samples of ASP and ATR with deliberate changes in optimized chromatographic conditions. The changes in the response of ASP and ATR as well as system suitability parameters were recorded. The parameters which changed include flow rate (± 0.1 mL), detection wavelength (± 2 nm), and organic component composition (±5%).

### Solution Stability

Solution stability of the standard as well as the sample was performed at benchtop (room temperature) and refrigeration temperature. The stability was checked by comparing the peak area of the standard and sample each time with the initial concentration.

### Limit of Detection and Quantification

The LOD and LOQ were determined from the standard deviation of the response and the slope of the calibration curve. The equation mentioned in the ICH guidelines was used for the determination of the LOD and LOQ.

## Results and Discussion

To carry out a detailed stability study of ATR and ASP in combination, it is essential to have a single stability-indicating assay method which can separate ATR and ASP along with all of its the degradation products from each other. Thus, a novel RP-HPLC method was developed and optimized using the Phenomenex C18 column (150×4.6 mm, 5μm particle size) and a mobile phase consisting of acetonitrile and water with 0.1% glacial acetic acid in the ratio of 50:50 v/v at a flow rate of 1.0 mL/min.

**Fig.3:**
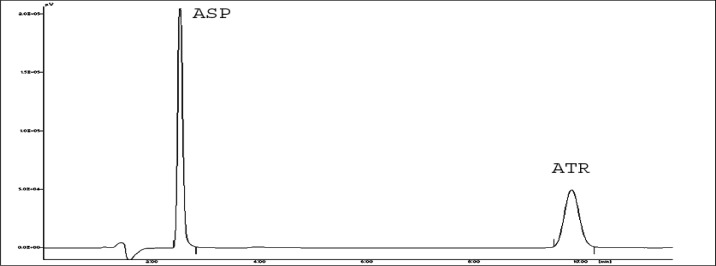
Representative HPLC chromatogram of standard ASP and ATR

The amount of ASP is 7.5 times more than ATR in the combined marketed dosage form. Therefore, to get the maximum response of analyte by this method, 246 nm was used as the detection wavelength for both drugs, which is the absorption maxima of ATR, and a promising response of ASP was also obtained. Fig. 3 shows the RP-HPLC chromatogram of ATR and ASP in combination with a retention time of 9.86 and 2.60 min respectively.

System suitability parameters of the developed method are summarized in Table 1.

### Validation of developed method

The developed method was successfully validated as per ICH guidelines by evaluating various validation parameters like linearity, repeatability, interday and intraday precision, LOD, and LOQ. Results of validation parameters are depicted in Table 2.

The mean % recovery values of ASP and ATR were obtained in the range of 99.0 ± 0.04 – 99.9 ± 0.27 and 99.0 ± 0.71 – 99.8 ± 0.41 which proves that the method is accurate. The results of the accuracy study are presented in Table 3.

The robustness of the methods was studied by analyzing the same samples of ASP and ATR with deliberate variation in the method parameters. It was found that there was no significant change in the system suitability parameters of both ASP and ATR. The results obtained in the changed parameters were within an acceptable range.

Specificity of the method was confirmed by checking the peak purity of ASP and ATR. The peak purity was found to be more than 990 for both ASP and ATR. The results obtained from the degradation studies showed that the developed stability-indicating assay method is specific towards ASP, ATR and their degradation products.

The prepared solution of ASP in diluent was found to be stable up to 6 hrs on the benchtop as well as after refrigeration. After 6 hrs, there was formation of the major degradation product of ASP i.e. salicylic acid. ATR was found to be stable for up to 24hrs in both benchtop as well as after refrigeration. In the case of ASP, a decrease in area after 6 hrs was more than 2% for both samples kept on the benchtop as well as samples kept in the refrigerator. Conversely for ATR, there was no significant decrease in the area after 24 hrs in both samples.

### Forced Degradation study

Degradation behavior of ASP as well as ATR was studied individually and in combination to check chemical compatibility in the presence of each other. Different stress conditions were employed as suggested by ICH guidelines.

For the stress degradation study, a selection of co-solvents was a challenging task for these poorly water-soluble drugs. ATR is very slightly soluble in water as well as acetonitrile. Both the drugs have good solubility in methanol, but due to the presence of the carboxylic acid functional group in both ATR and ASP, there is a chance that methyl ester may form in the drugs. Degradation of the drugs by the formation of methyl ester may lead to errors in the degradation study [27, 28]. DMSO is a comparatively inert solvent [29–30] and both the drugs have good solubility in it. Hence, it was selected as the co-solvent along with stressors for preparing solutions during the stress studies.

### ATR and ASP individually

ASP, being an ester moiety, is susceptible to acid, neutral, base, and oxidative degradation conditions. It primarily degrades into SA. SA was identified by analyzing standard SA under the same chromatographic conditions. Confirmation was done on the basis of retention time and PDA spectra. In photolytic and thermal stress conditions, ASP alone is stable. Results are in accordance with the reported studies [11, 23].

ATR alone is susceptible to acidic hydrolytic conditions and is converted initially into the major degradation product ATR lactone (ATR-DP). ATR-DP was identified by analyzing standard ATR-DP under the same chromatographic conditions. Confirmation was done on the basis of retention time and PDA spectra. Upon prolonged exposure, it further degraded into two additional degradation products other than ATR-DP. Stress degradation of ATR is also in accordance with the reported study [13, 23]. However, in neutral, base, oxidative, photolytic, and thermal stress conditions, ATR does not show any degradation. Table 4 summarizes the degradation behavior of ASP and ATR alone.

### ATR and ASP in the ratio of (1:1)

Except thermal degradation, no additional peak was observed in the HPLC chromate-grams of the stress degradation study of ATR and ASP in combination (1:1 ratio) compared to chromatograms of the individual degradation study in all conditions. This indicates that no additional degradation product formed other than SA and ATR-DP when ASP and ATR were present in combination. However, the percentage degradation of both drugs affected by the presence of each other, depending on the stress conditions, were employed for the study. The presence of ATR does not affect the stability of ASP in acidic hydrolytic conditions, as the observed percentage degradation was 24.24% and 20.28% in ASP alone and in combination, respectively.

In thermal degradation, three additional peaks of degradation products were observed. PDA spectra of all three degradation products correlated with the PDA spectra of ASP. This result indicates that additional degradation products may be generated from ASP. This is further supported by the highest difference in % degradation of ASP alone and in the presence of ATR under thermal degradation conditions.

Major influences of both drugs were observed on each other in the neutral, oxidative, and thermal degradation study. ATR was highly stable in the above stress conditions when present alone, but showed significant degradation when present in combination with ASP. Table 4 summarizes the percentage degradation of both drugs in various stress conditions.

### ATR and ASP in the ratio of 1:7.5 (formulation ratio)

**Fig. 4:**
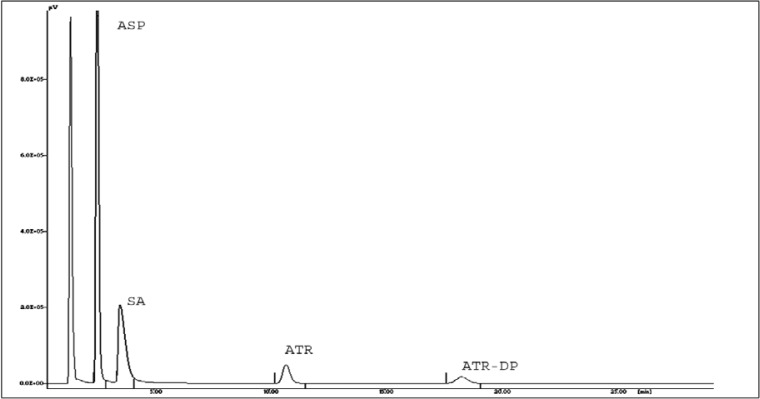
Representative HPLC Chromatogram for acid degradation of ATR and ASP in 1:7.5 combination.

**Fig. 5:**
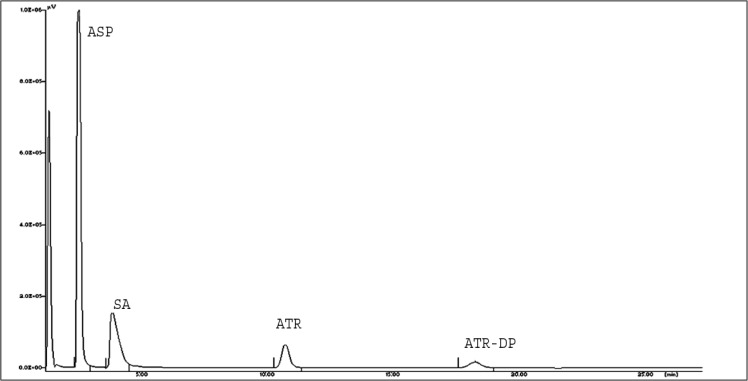
Representative HPLC Chromatogram for neutral degradation of ATR and ASP in 1:7.5 combination

**Fig. 6:**
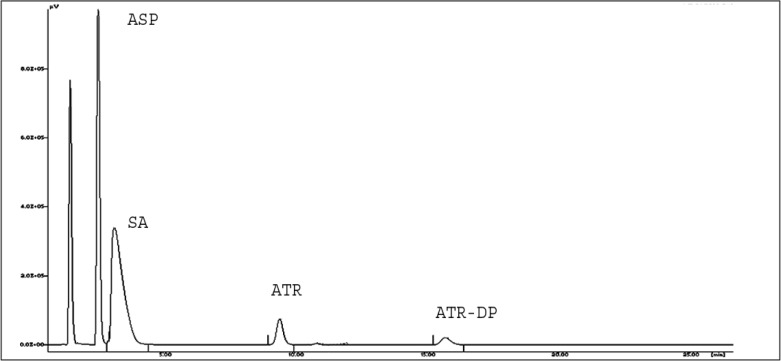
Representative HPLC Chromatogram for oxidative degradation of ATR and ASP in 1:7.5 combination.

It was decided to perform the stress degradation study of ATR and ASP in the ratio of 1:7.5 respectively, as both drugs are present in this ratio in the marketed fixed dose combination. Except for the thermal degradation study, no additional peak, other than SA and ATR-DP, was observed in the HPLC chromatogram of the mixture of ATR and ASP in 1:7.5 ratios. In acid degradation, ATR showed slightly higher degradation as compared to when it is present alone (Figure 4). But on increasing the amount of ASP up to 7.5 times, the degradation rate of ATR was increased particularly in the neutral, oxidative, and thermal stress degradation study as shown in figure 5, 6, and 7 respectively.

A summary of the degradation study of both drugs is furnished in Table 4. It was confirmed that both drugs showed a significant influence on the percentage degradation of each other if present together. From all of the results of the stress degradation studies, it can be summarized that both ASP and ATR can interact chemically and degrade if present in combination.

To get further scientific evidence, both of the drugs alone and in combination (in a ratio of 1:1 and 1:7.5, respectively, for ATR and ASP) were kept for one month stability studies at 40 °C and 75% RH. It was found that both ATR and ASP were stable if present alone. Conversely, significant degradation was observed if present in combination. ASP was found to be prone to degradation in the presence of ATR when both were in a 1:1 ratio. The percentage degradation of ATR was increased when combined with 7.5 times ASP (formulation ratio). The results indicated that the degradation of ATR is highly influenced by the amount of ASP present in the sample. Results of the one month stability study are furnished in Table 5.

## Conclusion

The degradation behavior of ASP and ATR was described at length in the present study. The stress study was considered as a tool to check the chemical incompatibility of both drugs in the presence of each other. From the stress degradation studies, it can be concluded that there is a significant difference in the % degradation of both of the drugs, alone and in combination with each other. This chemical instability of ASP and ATR is associated with the combined effect of temperature as well as humidity. Additionally, the combination ratio of ASP and ATR also influenced their degradation rate. Therefore, while formulating a fixed-dose combination of these two drugs, a proper formulation strategy is very important which can ensure the stability of both ASP and ATR in formulation.

## Figures and Tables

**Fig. 7:**
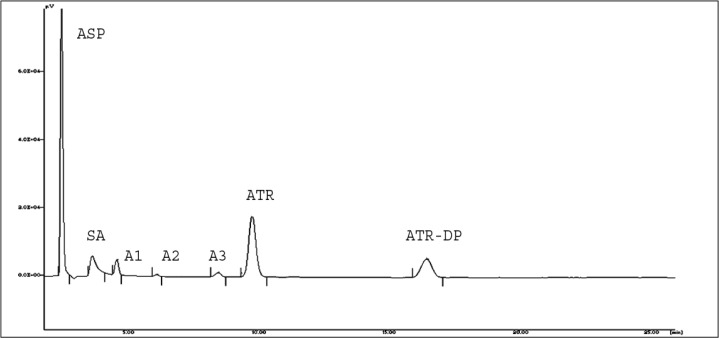
Representative HPLC Chromatogram for thermal degradation of ATR and ASP in 1:7.5 combination showing three additional degradation products (A1, A2, and A3).

**Tab. 1.** Results of System Suitability Parameters for ASP and ATR by the Proposed RP-HPLC Method

**Parameter**	**ASP**	**ATR**
Rt (min)	2.60	9.86
Asymmetry factor	1.24	1.17
Theoretical plates	4939	9509
Resolution	–	26.62

**Tab. 2.** Summary of Validation Parameters for the Proposed Method

**Parameter**	**ASP**	**ATR**
Linearity^a^ (μg/mL) (n=3)	1–80	1–60
Intercept (c)	−21282	−8019
Slope (m)	46251	36678
r^2^ value	0.999	0.999
LOD (μg/mL)	0.010	0.032
LOQ (μg/mL)	0.031	0.099
Precision		
Intraday % RSD (n=3)	0.19–1.08	0.18–0.85
Interday % RSD (n=3)	0.03–0.5	0.15–042
Repeatability % RSD (n=6)	0.94	0.78

a y = mx + c

**Tab. 3.** Results of Accuracy Studies of ASP and ATR by Proposed RP-HPLC Method

**Amount of drug taken (μg)**		**Amount of standard added(μg)**		**Mean % Recovery^a^ ± S.D**	

ASP	ATR	ASP	ATR	ASP	ATR
30	4	24	3.2	99.0± 0.04	99.8 ±0.41
30	4	30	4	99.0 ±0.10	99.2±0.31
30	4	36	4.8	99.6±0.27	99.0±0.71

a Average of three determinations.

**Tab. 4.** Summary of Stress Degradation Behavior for ASP and ATR

**Degradation Type**	**Degradation Condition**	**Sample Type**	**% Degradation observed for ASP**	**% Degradation observed for ATR**
Acid Hydrolysis	0.1 N HCl 3 Hrs reflux at 80 °C	ASP alone	24.25	–
		ATR Alone	–	29.6
		ASP:ATR(1:1) Ratio	20.28	38
		ATR:ASP(1:7.5)Ratio	32.26	36.40

Alkaline Hydrolysis	0.1 N NaOH 3 Hrs reflux at 80 °C	ASP alone	90	–
		ATR Alone	–	No Degradation
		ASP:ATR(1:1) Ratio	90	No Degradation
		ATR:ASP(1:7.5)Ratio	93	No Degradation

Neutral Hydrolysis	3Hrs reflux at 80 °C in water	ASP alone	19	–
		ATR Alone	–	No Degradation
		ASP:ATR(1:1) Ratio	39	6.28
		ATR:ASP(1:7.5)Ratio	25.61	27.22

Oxidative Stress	3% H_2_O_2_ at room temperature for 7 days	ASP alone	54.49	–
		ATR Alone	–	No Degradation
		ASP:ATR(1:1) Ratio	89	28
		ATR:ASP(1:7.5)Ratio	61	30

Thermal Stress	80 °C for 48 Hrs	ASP alone	No Degradation	–
		ATR Alone	–	No Degradation
		ASP:ATR(1:1) Ratio	39.61	38.57
		ATR:ASP(1:7.5)Ratio	15	50

Photolytic Stress	Exposed to sunlight for 12 hrs	ASP alone	No Degradation	No Degradation
		ATR Alone	No Degradation	No Degradation
		ASP:ATR(1:1) Ratio	No Degradation	No Degradation
		ATR:ASP(1:7.5)Ratio	No Degradation	No Degradation

**Tab. 5.** Summary of Degradation after One Month Stability Study at 40 °C and 75% RH.

**No**	**Sample Type**	**% Degradation observed for ASP**	**% Degradation observed for ATR**
1	ASP alone	No Degradation	–
2	ATR Alone	–	No Degradation
3	ASP:ATR(1:1) Ratio	18	31
4	ATR:ASP(1:7.5)Ratio	5	54
